# Assessment Value of Electromyography for Bortezomib-Related Peripheral Neuropathy

**DOI:** 10.2174/011570159X376896250624070328

**Published:** 2025-07-09

**Authors:** Yijun Shen, Zhen Zhang, Yuchen Liu, Siyuan Song, Tian Li, Jihong Dong, Guanru Niu

**Affiliations:** 1 Department of Neurology, Shanghai Geriatric Medical Center, Shanghai, China;; 2 Department of Rehabilitation, Shanghai Geriatric Medical Center, Shanghai, China;; 3 Department of Neurology, Zhongshan Hospital, Fudan University, Shanghai, China;; 4 Department of Geriatrics, Zhongshan Hospital, Fudan University, Shanghai, China;; 5 Department of Neuroscience, Baylor College of Medicine, Houston, TX, USA;; 6 Tianjin Key Laboratory of Acute Abdomen Disease-Associated Organ Injury and ITCWM Repair, Institute of Integrative Medicine of Acute Abdominal Diseases, Tianjin Nankai Hospital, Tianjin Medical University, Changjiang Avenue, Tianjin, 300100, China

**Keywords:** Electromyography, bortezomib, peripheral neuropathy, nerve conduction abnormalities, progression-free survival (PFS), neurotoxicity

## Abstract

**Introduction:**

This study investigates the relationship between National Cancer Institute Common Terminology Criteria (NCI-CTC) for grading bortezomib-induced peripheral neuropathy (BIPN) and objective motor/sensory nerve dysfunctions assessed by nerve conduction studies (NCS). It also evaluates the correlation between specific nerve conduction abnormalities and progression-free survival (PFS).

**Methods:**

Thirty-three patients with multiple myeloma developing peripheral neuropathy during bortezomib treatment were enrolled. Participants were grouped based on NCI-CTC toxicity scores (< 2, n=17; ≥ 2, n=16). Comprehensive NCS were performed, assessing compound muscle action potentials (CMAP), motor conduction velocities (MCV), sensory nerve action potentials (SNAP), and sensory conduction velocities (SCV) across ulnar, median, tibial, peroneal, sural, and superficial peroneal nerves. Correlation analyses were used to examine the association between NCS parameters and PFS.

**Results:**

Patients with higher NCI-CTC grades (≥ 2) exhibited significant reductions in motor and sensory nerve conduction parameters. Notably, the peroneal nerve showed significant decreases in CMAP (*p=*0.0059) and MCV (*p=*0.0223). The superficial peroneal nerve displayed a significant reduction in SCV (*p=*0.0189). A strong positive correlation was found between median nerve SNAP and longer PFS (r=0.558, *p=*0.001).

**Discussion:**

The findings indicate that higher clinical grades of BIPN (NCI-CTC ≥ 2) are associated with objective neurophysiological evidence of worsened nerve function, with the peroneal nerve being particularly affected. The correlation between median nerve SNAP and PFS suggests that NCS parameters could potentially serve as prognostic markers in patients with BIPN.

**Conclusion:**

Bortezomib-induced neurotoxicity leads to significant impairments in both motor and sensory nerve conduction. Median nerve SNAP shows promise as a predictor for PFS, underscoring the potential value of NCS in monitoring neurotoxicity and guiding clinical management in patients receiving bortezomib.

## 1 INTRODUCTION

Multiple myeloma (MM) is a malignant disorder of plasma cells, accounting for approximately 1% of all cancers and 12% of hematological malignancies worldwide [1]. The disease is characterized by the clonal proliferation of abnormal plasma cells in the bone marrow, leading to immunodeficiency, renal dysfunction, anemia, and bone lesions [2]. Over the past two decades, treatment options for MM have greatly expanded with the introduction of novel therapies, including proteasome inhibitors, immunomodulatory drugs, and monoclonal antibodies [3]. Among these, bortezomib, a first-in-class proteasome inhibitor, has shown remarkable efficacy in prolonging survival rates for both newly diagnosed and relapsed/refractory MM patients [4]. Bortezomib-induced Peripheral Neuropathy (BIPN) is thought to arise from neurotoxic effects on the dorsal root ganglia and peripheral nerves, potentially caused by bortezomib-related mitochondrial damage, oxidative stress, and impaired axonal transport, although the precise mechanisms are still uncertain [5-8]. Despite its clinical success, bortezomib is often associated with significant adverse effects, the most debilitating of which is peripheral neuropathy [9-12]. The reported incidence of overall-grade BIPN in clinical trials ranges from 15% to 57.2%, with a median of 33.9% (95% confidence interval: 29.9-38.5%). In contrast, the occurrence of severe grade 3-4 BIPN events varies between 1% and 30%, with a median of 8.1% (95% confidence interval: 6.9-9.4%), depending on study design, assessment criteria, and patient demographics [13-19]. These differences depend on the specific studies, how BIPN is measured, and the type of patients involved [16, 17, 20, 21]. The onset of BIPN typically follows a cumulative dose-response relationship, with sensory neuropathy being more common than motor involvement [22]. Importantly, BIPN is dose-limiting, often necessitating dose reductions or discontinuation, which can compromise the therapeutic efficacy of the treatment [23]. The clinical manifestations of BIPN include numbness, tingling, and a burning sensation in the extremities, primarily affecting the hands and feet in a distal-to-proximal distribution. This “glove and stocking” pattern is characteristic of length-dependent axonal damage. In severe cases, some patients develop motor issues, including significant symptoms like muscle weakness, muscle wasting, foot drop on one or both sides, and reduced reflexes [24, 25]. The increasing incidence of BIPN due to rising global tumor rates and longer survival times, along with its detrimental impact on chemotherapy and quality of life, underscores the urgent need for research on its mechanisms and therapies, as there are currently no American Society of Clinical Oncology (ASCO)-recommended treatments [26]. Accurate and standardized assessment of BIPN is crucial for diagnosis, epidemiological monitoring, and treatment decisions. Current evaluation methods include clinical examinations, neurophysiological tests (*e.g*., nerve conduction studies, quantitative sensory testing, and skin biopsy), and patient-reported outcome measures [27, 28]. However, the lack of standardized diagnostic criteria and underreporting by patients often leads to an underestimation of BIPN incidence and severity [11, 29, 30]. In this context, electrophysiological studies have emerged as valuable tools for assessing the severity and progression of peripheral neuropathy. Nerve Conduction Studies (NCS) allow for the quantitative evaluation of nerve conduction and muscle response, providing insight into the extent of nerve damage. By measuring the amplitude, latency, and conduction velocity of peripheral nerves, NCS can help differentiate between demyelinating and axonal neuropathies. Moreover, it can detect subclinical neuropathy, offering a means for early intervention before irreversible damage occurs. Several studies have highlighted the role of NCS in monitoring Chemotherapy-Induced Peripheral Neuropathy (CIPN), demonstrating its potential in guiding treatment decisions and preventing long-term neurological complications.

This study aims to investigate the relationship between nerve conduction abnormalities and the clinical severity of BIPN in MM patients undergoing bortezomib treatment. Specifically, this study proposes the following hypotheses: 1). High-risk patient characteristics: Certain demographic and clinical factors are associated with increased BIPN severity, providing a basis for individualized chemotherapy regimen adjustments and early intervention. Identifying susceptible populations could optimize treatment strategies, such as avoiding highly neurotoxic agents or incorporating neuroprotective interventions. 2). Correlation between NCS parameters and National Cancer Institute Common Terminology Criteria (NCI-CTC) grading: NCS could serve as an objective assessment tool for chemotherapy-induced neurotoxicity, reducing reliance on subjective symptom reporting and assisting clinicians in dynamically adjusting treatment regimens. 3). Prognostic value of NCS indicators: If NCS parameters independently predict progression-free survival (PFS), this would suggest that neurological damage correlates with disease progression or treatment tolerance. If validated, NCS could serve as a low-cost, reproducible prognostic biomarker to optimize risk stratification and guide personalized treatment strategies.

## 2 MATERIALS AND METHODS

### 2.1 Participants Recruitment and Criteria

Adult patients diagnosed with MM based on the International Myeloma Working Group Criteria and admitted to the Hematology Department of Zhongshan Hospital, affiliated with Fudan University, were consecutively enrolled in this study. All participants underwent treatment with bortezomib and subsequently developed symptoms of peripheral neurotoxicity. Each patient provided written informed consent prior to participation. The study protocol was reviewed and approved by the Ethics Committee of Zhongshan Hospital, Fudan University (Ethical Approval Number: B2016-144R, Approval Date: 8 December 2016).

Inclusion Criteria: 1) Adult patients of both genders, aged between 18 and 75 years, to ensure a broad representation of the population while excluding pediatric and elderly patients who may have additional risk factors for neuropathy. 2) Diagnosis of MM: Patients must have a confirmed diagnosis of MM based on the criteria established by the International Myeloma Working Group (IMWG) or the World Health Organization (WHO). Diagnosis should be verified through clinical, laboratory, and imaging findings. 3) Treatment Protocol: Patients must be undergoing treatment with bortezomib as the sole chemotherapy agent, without any prior exposure to other chemotherapeutic drugs, immunomodulatory agents, or corticosteroids as primary treatment for MM. 4) Patients must provide written informed consent to participate in the study after receiving a detailed explanation of the study's objectives, potential risks, and benefits.

Exclusion Criteria: 1) Patients with pre-existing neuropathic conditions, including diabetic neuropathy, hereditary neuropathies, CIDP, or any other peripheral neuropathy of known etiology, will be excluded to prevent confounding factors in assessing bortezomib-induced neuropathy. 2) Other Hematological Disorders or Prior Chemotherapy: Patients with other hematological malignancies (*e.g*., leukemia, lymphoma, myelodysplastic syndromes) or those who have received any form of prior chemotherapy, including alkylating agents, immunotherapy, or radiotherapy, will be excluded to ensure that neuropathic symptoms are solely attributable to bortezomib treatment. 3) Pre-existing Neurological Conditions and Lifestyle Factors: Patients with pre-existing neurological disorders such as multiple sclerosis, Parkinson’s disease, stroke, or epilepsy will be excluded. Additionally, patients with a history of severe vitamins B1 (thiamine), B6 (pyridoxine), or B12 (cobalamin) deficiencies will be excluded, as these conditions can independently contribute to neuropathic symptoms and confound study results.

**Fig. (1):**
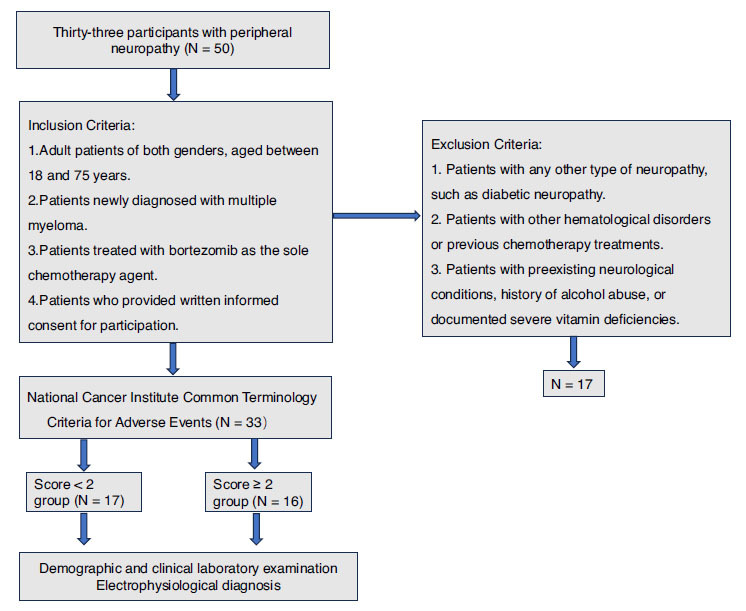
Flow chart of this article. Selection of patients with multiple myeloma, which shows inclusion and exclusion criteria as well as subgroup assignment and patient numbers.

The recruitment process involved screening eligible patients from hospital records and outpatient visits. Patients meeting the inclusion criteria were approached by the research team, provided with study details, and given the opportunity to ask questions before consenting to participate. Those who met the exclusion criteria were removed from consideration. A total of 33 patients were enrolled following this process (Fig. **1**).

### 2.2 Demographic Information

Detailed demographic and clinical information was collected from all participants, including age, sex, and specific risk factors relevant to neurotoxicity and multiple myeloma, such as duration of MM diagnosis, comorbidities, and other potential contributing factors (*e.g*., smoking history, family medical history, and baseline kidney function).

### 2.3 Neuropsychological Assessment

Neuropsychological assessments were conducted to evaluate the impact of peripheral neurotoxicity on participants. In this study, the NCI-CTC was used, a clinician-based grading scale, to assess the severity of peripheral neuropathy, as it remains the most widely utilized tool for BIPN evaluation [31, 32]. The NCI-CTC system (http://www.eortc.be/services/doc/ctc) evaluates sensory and motor neuropathy on a five-grade scale as follows:

• Grade 1: Asymptomatic; loss of deep tendon reflexes or paresthesia (including tingling), but not interfering with function.
• Grade 2: Moderate symptoms; limiting instrumental activities of daily living (ADLs).
• Grade 3: Severe symptoms; limiting self-care ADLs.
• Grade 4: Life-threatening consequences; urgent intervention indicated.
• Grade 5: Death related to neuropathy.

Each patient’s BIPN severity was assessed by an experienced neurologist and oncologist based on clinical symptoms and neurological examination findings.

### 2.4 Electromyography Acquisition

Electrophysiological data were obtained from the Electromyography Department at Zhongshan Hospital using EMG (electromyography) equipment (Natus, America). The following neurophysiological measures were recorded:

#### 2.4.1 Nerve Conduction Studies (NCS)

These studies were conducted to measure the speed and strength of electrical signals in peripheral nerves, using a Medelec Synergy electromyography machine. For motor nerves, the right median and right peroneal nerves were evaluated. For sensory nerves, the right median and bilateral sural nerves were assessed.

#### 2.4.2 Motor Nerves

Compound muscle action potentials (CMAPs) were recorded from the abductor pollicis brevis following stimulation of the right median nerve. Similarly, motor conduction in the right peroneal nerve was assessed, and CMAPs were recorded from the extensor digitorum brevis.

Motor conduction velocity (MCV) was determined by measuring the distance between the stimulation sites and dividing it by the difference in latency. For the median nerve, proximal and distal stimulations were applied at the elbow and wrist, respectively. For the peroneal nerve, stimulations were delivered at the fibular head and ankle.

#### 2.4.3 Sensory Nerves

Sensory nerve action potentials (SNAPs) were recorded from the second digit following antidromic stimulation of the right median nerve at the wrist. Additionally, bilateral sural SNAPs were measured following antidromic stimulation at the lateral malleolus, with the stimulation delivered 14 cm proximally at the mid-calf. Sensory conduction velocity (SCV) was calculated by dividing the distance between the stimulation and recording sites by the difference in latency. For the median nerve, the distance between the wrist and the recording electrode on the second digit was used. For the sural nerve, the distance from the lateral malleolus to the mid-calf stimulation site was measured. To ensure accurate conduction measurements, skin temperature was maintained at or above 32.0°C near the site of nerve stimulation. If required, heating pads were applied to regulate temperature.

### 2.5 Statistical Analysis

Statistical analyses were performed to compare clinical characteristics and nerve conduction parameters (*e.g*., CMAP, MCV, SNAP, SCV) between NCI-CTC grade subgroups. Normality of data distribution was assessed using the Shapiro-Wilk test (*p* > 0.05), and homogeneity of variance was validated *via* Levene’s test (*p* > 0.05). For variables meeting both assumptions, independent two-sample t-tests were applied to evaluate group means, with effect sizes reported as Cohen’s d. Non-normally distributed or heteroscedastic data were analyzed using Mann-Whitney U tests, supplemented by Hodges-Lehmann median differences for effect size quantification. Associations between nerve conduction parameters and PFS were examined through two-tailed Pearson’s correlation (normally distributed data) or Spearman’s rank correlation (non-normal/ordinal data), with coefficients (r) and 95% confidence intervals (CI) calculated. Statistical significance was defined as a two-sided *p*-value < 0.05. Continuous variables were reported as mean ± standard deviation (SD) or median (interquartile range, IQR) based on distribution characteristics. All statistical analyses were generated using SPSS (version 29.0.2.0, IBM Corp.) and GraphPad Prism (version 9.0.0, GraphPad Software).

## 3 RESULTS

### 3.1 Demographic and Clinical Characteristics of Participants between Different NCI-CTC Groups

Table **1** presents the demographic and clinical characteristics of 33 participants, divided into two groups based on NCI-CTC (NCI-CTC < 2 and NCI-CTC ≥ 2). The average age of the participants was 61.09 years, with no significant difference between the two groups (*p=*0.9619). Regarding gender, 60.6% of the participants were male and 39.4% were female. Although a higher proportion of males was observed in the NCI-CTC < 2 group (70.6%) compared to the NCI-CTC ≥ 2 group (50.0%), the gender distribution did not show statistical significance (*p=*0.2960). The body mass index (BMI) showed a slight difference between the groups, with a mean of 24.54 kg/m^2^ in the NCI-CTC < 2 group and 23.34 kg/m^2^ in the NCI-CTC ≥ 2 group, but this difference was not statistically significant (*p=*0.3055). In terms of immunoglobulin types, 54.5% of participants had IgG, with 64.7% in the NCI-CTC < 2 group and 43.8% in the NCI-CTC ≥ 2 group. A higher proportion of patients with IgA was found in the NCI-CTC ≥ 2 group (31.3%), but there was no significant difference between the groups (*p=*0.3419). Regarding light chain types, the distribution was similar between the groups, with 52.9% of the NCI-CTC < 2 group and 56.3% of the NCI-CTC ≥ 2 group having the λ (lambda) type. The κ (kappa) type was more prevalent in the NCI-CTC < 2 group (41.1% *vs*. 31.3%), though this difference was not significant (*p=*0.7273). For International Staging System (ISS) stages, both groups had a similar distribution, with approximately half of the patients in stage 3 (*p=*0.6949). The time from disease onset to electromyography (EMG) was not significantly different between the groups (*p=*0.2935), and the duration of treatment to NCS was also comparable (*p=*0.4448). The proportion of patients receiving preventative neuroprotective treatments was similar between the groups (*p=*0.6562). Overall, there were no statistically significant differences between the groups for any of the analyzed variables, indicating that NCI-CTC scores may not be strongly associated with the baseline demographic and clinical characteristics of the patients.

### 3.2 Amplitude and Conduction Velocity of Motor Nerves in Different NCI-CTC Grades

Table **2** compares the CMAP amplitude and MCV of four motor nerves between two groups of patients based on their NCI-CTC toxicity grades: those with a score < 2 and those with a score ≥ 2. Across all nerves, patients with a higher toxicity score (≥ 2) generally showed lower CMAP amplitudes and slower MCV, indicating reduced motor nerve function. In the ulnar nerve, both CMAP (*p=*0.2967) and MCV (*p=*0.4006) showed slight decreases in the higher toxicity group, but the differences were not statistically significant. Similarly, the tibial nerve displayed non-significant differences in CMAP (*p=*0.4705) and MCV (*p <* 0.0001), though the latter reached statistical significance, indicating significantly slower motor conduction in this nerve among patients with higher toxicity. The median nerve had a borderline significant reduction in CMAP (*p=*0.0746) and a statistically significant reduction in MCV (*p=*0.0360), suggesting that motor conduction velocity is more affected by higher toxicity levels in this nerve. The most significant findings were observed in the peroneal nerve, where both CMAP (*p=*0.0059) and MCV (*p=*0.0223) showed substantial decreases in the score ≥ 2 group, with both measures reaching statistical significance. These results indicate that higher NCI-CTC toxicity grades are associated with more pronounced impairments in motor nerve function, particularly in the tibial and peroneal nerves. The significant reductions in motor conduction velocity in these nerves suggest that they are more vulnerable to damage in patients with greater toxicity, making them potentially useful markers for assessing motor nerve dysfunction in these individuals.

### 3.3 Amplitude and Conduction Velocity of Sensory Nerves in Different NCI-CTC Grades

Table **3** compares the SNAP amplitude and SCV of four different sensory nerves between two groups of patients classified by their NCI-CTC toxicity grade: those with a score < 2 and those with a score ≥ 2. Across all nerves, patients with higher NCI-CTC scores (≥ 2) generally exhibited lower SNAP amplitudes and slower conduction velocities, indicating poorer nerve function in this group. The ulnar nerve showed a modest decline in both SNAP (*p=*0.3599) and SCV (*p=*0.0919) in the score ≥ 2 group, but the results were not statistically significant. Similarly, the median nerve demonstrated reductions in SNAP (*p=*0.1928) and SCV (*p=*0.1304), though again, without statistical significance. In contrast, the sural nerve had a notable decline in SCV in the score ≥ 2 group (*p=*0.0652), approaching significance. The most significant findings were observed in the superficial peroneal nerve, where both SNAP (*p=*0.0584) and SCV (*p=*0.0189) showed considerable reductions in the higher NCI-CTC score group, with the SCV reaching statistical significance (*p <* 0.05).

### 3.4 Correlation between NCS Indexes and PFS of Patients

The table presents the correlation between various NCS indices and the PFS of patients (Table **4**). The results show that the median nerve SNAP has the strongest positive correlation with PFS, with a correlation coefficient (r) of 0.558 and a statistically significant *p*-value of 0.001. This suggests that patients with better median nerve SNAP tend to experience longer progression-free survival.

Additionally, the Median nerve SCV also exhibits a moderate positive correlation with PFS (r = 0.366, *p=*0.043), indicating that sensory conduction velocity in the median nerve is significantly associated with PFS. Similarly, the Superficial Peroneal nerve SNAP has a positive correlation with PFS (r = 0.384, *p=*0.030), which is also statistically significant.

The Ulnar nerve SNAP and Sural nerve SNAP display moderate positive correlations with PFS (r = 0.334, *p=*0.067 and r = 0.343, *p=*0.055, respectively), though these results are not statistically significant at the 0.05 level, suggesting that their associations with PFS might be weaker or less certain.

## 4 DISCUSSION

### 4.1 Main Interpretation

This study, along with several large clinical trials, did not clearly establish a relationship between BIPN and age or gender [19, 33]. The distribution of immunoglobulin types, light chain types, ISS stage, and disease duration prior to NCS testing, as well as treatment duration before NCS, does not show significant differences between the two groups. Most patients received preventative neuroprotectants, but this factor also lacked statistical significance. This suggests that MM-related BIPN may be more driven by genetic factors, treatment, or the disease itself rather than demographic factors. A recent genetic study supports this, showing that BIPN is more likely to occur in patients with lower MTHFR mRNA expression [34]. But some studies have reported different findings from ours. For example, in MM patients, those aged ≥70 have more severe baseline PN compared to those under 70. Additionally, excessive λ light chain production may damage peripheral nerves [35]. However, due to our small sample size, further investigation into the relationship between these indicators and BIPN will require the collection and analysis of a larger patient cohort.

Neuropathy is most effectively evaluated through patient-reported symptom severity, objective clinical sign scales, and neurophysiological testing [15]. This study found that MM patients primarily exhibit sensory nerve damage as the main clinical symptom; however, NCS indicates that a significant portion also has motor nerve damage. There were statistically significant differences in the MCV of the median and common peroneal nerves, while the CMAP of the common peroneal nerve also showed a statistically significant difference. To further contextualize the findings of this study within the existing literature, the results were compared with studies examining BIPN and other forms of chemotherapy-induced neuropathy. Prior research has indicated that sensory nerve involvement, particularly in the sural nerve, serves as an early marker of neurotoxicity [33, 36-39]. Several electrophysiological tests involving four to five patients showed that BIPN can present with demyelinating features and axonal degeneration, as confirmed by nerve biopsy [40, 41]. In a study of 120 CIPN patients, where nearly 90% reported neuropathic symptoms, CIPN was more commonly associated with ulnar and tibial nerve damage in this study population [42]. The combination therapy of bortezomib and thalidomide significantly decreases sural sensory nerve action potentials and motor nerve action potentials [40]. One study's conclusions differ from ours, showing no correlation between symptom severity and bortezomib dosage or nerve conduction studies [14].

Furthermore, motor nerve involvement has significant clinical implications beyond functional decline; whether motor nerves are affected is a key factor influencing patients' main neurological function scores. It is well-established that motor dysfunction in BIPN patients can lead to reduced mobility and an increased risk of falls, which significantly affects patients' quality of life. The findings from this study on MCV reduction in the peroneal nerve, in particular, are consistent with reports indicating a strong association between peroneal nerve impairment and lower extremity weakness. Bortezomib is typically thought to affect small myelinated fibers primarily; however, this study found that it also impacts large sensory fibers. The four sensory nerves test indicates that the superficial peroneal nerve is more susceptible to damage or dysfunction in the context of increased toxicity, making it a potentially valuable marker for assessing peripheral nerve damage in these patients. Although the SNAP and SCV of the other three nerves did not reach statistical significance, they still exhibited a marked decline. This could reflect the widespread impact of the disease on multiple nerves, with varying degrees of involvement across different nerves. This observation is consistent with previous research indicating that sensory fibers, particularly in the sural and peroneal nerves, are more likely to exhibit early subclinical signs of damage in neuropathies, often preceding overt motor symptoms. Abnormal sural nerve sensory nerve action potentials were found in 86% of patients, with the mean SNAP amplitude falling below the 3% lower limit of normal in 19 out of 22 patients [11].

This study also highlights the importance of considering both the specificity and sensitivity of NCS in predicting BIPN progression. While previous reports suggest variability in NCS thresholds across different neuropathies, the data indicate that the median nerve SNAP amplitude could serve as a robust predictor of clinical deterioration in BIPN patients, underscoring the potential for integrating NCS into routine monitoring protocols for patients receiving neurotoxic chemotherapy. The strong correlation between median nerve SNAP amplitude and PFS (r=0.558, *p=*0.001) further highlights EMG/NCS's prognostic potential. Importantly, the results suggest that EMG/NCS parameters may provide a non-invasive and quantifiable method to predict BIPN severity, complementing other clinical assessment tools. While prior studies have focused on composite NCS scores, the findings emphasize that single-nerve monitoring may offer comparable diagnostic value with improved feasibility.

### 4.2 Clinical Implications and Recommendations

Given the high prevalence of BIPN, regular NCS should be integrated into the clinical management of patients receiving neurotoxic chemotherapy. Early detection of neuropathy is critical for implementing timely interventions that can alleviate symptoms and preserve quality of life. Integrating pharmacological treatments such as duloxetine or gabapentin alongside non-pharmacological approaches like physical therapy and acupuncture may benefit patients with CIPN. Exercise has been shown to reduce CIPN symptoms in cancer patients undergoing taxane-, platinum-, or vinca alkaloid-based chemotherapy, particularly in older patients [43, 44]. Understanding the risk factors associated with CIPN can aid in identifying patients at higher risk for these complications, facilitating targeted interventions. Future research should also explore neuroprotective strategies and rehabilitation therapies aimed at mitigating nerve damage severity, particularly in high-toxicity groups. NCS are crucial for identifying subclinical neuropathies before they manifest as debilitating symptoms. Furthermore, the identification of the peroneal nerve as particularly vulnerable to neurotoxic damage suggests that these nerves could serve as focal points for monitoring CIPN progression in cancer patients.

## CONCLUSION

In conclusion, this study demonstrates significant motor and sensory nerve conduction impairments in patients with higher NCI-CTC grades. The observed correlations between NCS parameters and PFS highlight the potential prognostic value of neurophysiological assessments in chemotherapy-induced neurotoxicity. These findings add to the growing body of literature on the clinical utility of NCS in monitoring neurotoxicities. However, given the study’s limitations, including sample size and potential selection bias, caution is warranted in extrapolating these results to broader patient populations. Additionally, the inclusion criteria and potential selection bias may limit the applicability of the findings to broader populations. Larger, multi-center studies are necessary to confirm the associations between NCS parameters and PFS in broader populations.

Furthermore, as nerve conduction studies primarily assess large myelinated fibers, the impact on small fiber neuropathy remains insufficiently explored, which may contribute to an incomplete understanding of BIPN pathophysiology. Additionally, the study's cross-sectional design prevents definitive conclusions about causality between nerve conduction impairments and long-term outcomes. Future research should focus on validating these findings through larger prospective studies, integrating multi-modal diagnostic approaches, and exploring neuroprotective interventions aimed at mitigating chemotherapy-induced nerve damage. These efforts will be critical for refining risk stratification and optimizing patient management in clinical practice.

## Figures and Tables

**Table 1:** Demographic and clinical characteristics of participants between different NCI-CTC groups.

**-**	**Total Series** **n=33**	**NCI-CTC < 2** **n=17 (51.5%)**	**NCI-CTC ≥ 2** **n=16 (48.5%)**	***p*-value**
**Age, years**	61.09 ± 10.35	61.18 ± 9.36	61.00±10.61	0.9619
**Gender**	-	-	-	-
Male	20 (60.6)	12 (70.6)	8 (50.0)	0.2960
Female	13 (39.4)	5 (29.4)	8 (50.0)	-
**BMI, kg/m^2^**	23.99 ± 3.19	24.54 ± 3.25	23.34 ± 3.09	0.3055
**Immunoglobin type**	-	-	-	-
IgG	18 (54.5)	11 (64.7)	7 (43.8)	0.3419
IgA	7 (21.2)	2 (11.8)	5 (31.3)	-
NA	8 (24.2)	4 (23.5)	4 (25.0)	-
**Light chain type**	-	-	-	-
λ	18 (54.5)	9 (52.9)	9 (56.3)	0.7273
κ	12 (36.4)	7 (41.1)	5 (31.3)	-
NA	3 (9.1)	1 (5.9)	2 (12.5)	
**ISS stage**	-	-	-	-
1	11 (33.3)	4 (23.5)	2 (12.5)	0.6949
2	6 (18.2)	5 (29.4)	6 (37.5)	-
3	16 (48.5)	8 (47.1)	8 (50.0)	-
**Disease duration to EMG, months**	7.00 (5.00, 12.00)	8.00 (5.50, 12.00)	6.50 (5.50, 12.50)	0.2935
**Treatment duration to EMG, months**	6.00 (4.50, 10.00)	6.00 (4.50, 12.00)	5.50 (4.25, 7.75)	0.4448
**No. of treatment cycles**	5.00 (4.00, 6.00)	5.00 (4.00, 7.75)	5.00 (3.25, 5.00)	0.2315
**Preventative neuroprotectant**	27 (81.9)	13 (76.5)	14 (87.5)	0.6562
**PFS, months**	37.00 (20.75, 56.75)	41.00 (12.25, 74.25)	34.00 (26.75, 52.25)	0.4731

**Note**: All data are presented as n (%), mean ± SD, or median (IQR) based on data types and distribution characteristics. All doses were administered subcutaneously, with a dosage of 1.3 mg/m^2^.

**Table 2:** Amplitude and conduction velocity of motor nerves in different NCI-CTC grades.

**-**	**NCS Index**	**NCI-CTC Grades**		**Test Statistic†**	**Effect Size (95% CI)#**	***p-*value**
		**Score < 2 (n = 17)**	**Score ≥ 2 (n = 16)**			
Ulnar Nerve	CMAP, mv	8.06 ± 3.76	6.70 ± 3.34	*t* = 1.06	0.38 (-0.33, 1.09)	0.2967
	MCV, m/s	52.78 ± 6.17	50.44 ± 5.69	*U* = 98.00	1.80 (-2.30, 5.90)	0.4006
Median Nerve	CMAP, mv	8.61 ± 4.11	6.14 ± 4.26	*U* = 80.50	2.14 (-0.40, 6.75)	0.0746
	MCV, m/s	53.57 ± 2.90	50.38 ± 4.65	*U* = 72.50	2.15 (0.25, 5.30)	**0.0360**
Tibial Nerve	CMAP, mv	7.11 ± 4.16	5.97 ± 4.51	*U* = 88.00	1.84 (-1.90, 4.78)	0.4705
	MCV, m/s	42.54 ± 4.73	41.37 ± 2.57	*t* = 0.82	0.30 (-0.43, 1.03)	0.4207
Peroneal Nerve	CMAP, mv	4.02 ± 2.38	2.00 ± 1.67	*U* = 61.00	2.21 (0.34, 3.60)	**0.0059**
	MCV, m/s	43.38 ± 4.81	37.11 ± 10.48	*U* = 73.00	4.10 (0.90, 7.10)	**0.0223**

**Note**: †Test statistics: *t* for parametric comparisons (unpaired t-test), *U* for non-parametric comparisons (Mann-Whitney U test).

#Effect sizes are reported as Cohen’s d for unpaired t-tests and as Hodges-Lehmann median differences (same units as NCS Index) for Mann-Whitney U tests.

**Abbreviations**: NCS: nerve conduction studies; CMAP: compound motor active potentials; MCV: motor conduction velocity; NCI-CTC: National Cancer Institute-Common Toxicity Criteria; CI: confidence interval. The bold *p*-values mean a numerical value of *p <* 0.05.

**Table 3:** Amplitude and conduction velocity of sensory nerves in different NCI-CTC grades.

**-**	**NCS Index**	**NCI-CTC Grades**		**Test Statistic†**	**Effect Size (95% CI)#**	***p-*value**
		**Score < 2 (n = 17)**	**Score ≥ 2 (n = 16)**			
Ulnar Nerve	SNAP, μv	6.88 ± 4.86	5.35 ± 4.47	*t* = 0.93	0.33 (-0.37, 1.02)	0.3599
	SCV, m/s	52.14 ± 7.10	45.21 ± 12.75	*U* = 83.00	3.63 (-0.65, 8.90)	0.0919
Median Nerve	SNAP, μv	11.66 ± 10.06	7.03 ± 5.99	*U* = 93.00	2.93 (-1.50, 8.60)	0.1928
	SCV, m/s	53.59 ± 5.69	41.99 ± 21.39	*U* = 87.50	4.25 (-1.00, 10.20)	0.1304
Sural Nerve	SNAP, μv	8.11 ± 6.32	5.53 ± 5.37	*U* = 101.00	1.90 (-1.50, 6.35)	0.2102
	SCV, m/s	40.24 ± 17.15	27.27 ± 21.97	*U* = 85.00	3.98 (0.00, 36.70)	0.0652
Superficial Peroneal Nerve	SNAP, μv	8.51 ± 7.69	4.18 ± 4.20	*U* = 83.50	2.96 (0.00, 7.15)	0.0584
	SCV, m/s	40.89 ± 12.20	26.74 ± 19.68	*U* = 71.50	7.55 (0.80, 30.10)	**0.0189**

**Note**: †Test statistics: *t* for parametric comparisons (unpaired t-test), *U* for non-parametric comparisons (Mann-Whitney U test).

#Effect sizes are reported as Cohen’s d for unpaired t-tests and as Hodges-Lehmann median differences (same units as NCS Index) for Mann-Whitney U tests.

**Abbreviations**: NCS: nerve conduction studies; SNAP: sensory nerve action potential; SCV: sensory conduction velocity; NCI-CTC: National Cancer Institute-Common Toxicity Criteria; CI: confidence interval. The bold *p*-values mean a numerical value of *p <* 0.05.

**Table 4:** Correlation between NCS indexes and PFS of patients.

**-**	**Correlation Coefficient (r)**	**95% CI**	***p-*value**
Ulnar nerve SNAP	0.334	-0.024 to 0.615	0.067
Median nerve SNAP	0.558	0.254 to 0.762	0.001
Median nerve SCV	0.366	0.003 to 0.644	0.043
Sural nerve SNAP	0.343	-0.006 to 0.618	0.055
Superficial Peroneal nerve SNAP	0.384	0.040 to 0.646	0.030

**Abbreviations**: NCS: nerve conduction studies; SNAP: sensory nerve action potential; SCV: sensory conduction velocity; NCI-CTC: National Cancer Institute-Common Toxicity Criteria; CI: confidence interval. Parameters with *p*-values less than 0.1 are listed in the table. Two-tailed Pearson/Spearman regression analysis was used based on the distribution of each parameter.

## Data Availability

Data are available on https://www.jianguoyun.com/p/DVeFRbgQuaiFChiV_-EFIAA.

## LIST OF ABBREVIATIONS

ASCO: American Society of Clinical Oncology
BIPN: Bortezomib-induced Peripheral Neuropathy
BMI: Body Mass Index
CIDP: Chemotherapy-induced Peripheral Neuropathy
CMAP: Compound Muscle Action Potential
EMG: Electromyography
IgG: Immunoglobulin G
ISS: International Staging System
MCV: Motor Conduction Velocity
MM: Multiple Myeloma
NCI-CTC: National Cancer Institute Common Terminology Criteria
NCS: Nerve Conduction Studies
PFS: Progression-free Survival
SCV: Sensory Conduction Velocity
SNAP: Sensory Nerve Action Potential

## STANDARDS OF REPORTING

STROBE guidelines were followed.
